# New Autophagy-Ferroptosis Gene Signature Predicts Survival in Glioma

**DOI:** 10.3389/fcell.2021.739097

**Published:** 2021-11-15

**Authors:** Liwei Zhou, Zhengye Jiang, Zhongjie Shi, Wenpeng Zhao, Zhenwei Lu, Yuanyuan Xie, Bingchang Zhang, Hanwen Lu, Guowei Tan, Zhanxiang Wang

**Affiliations:** ^1^ Department of Neurosurgery, Xiamen Key Laboratory of Brain Center, The First Affiliated Hospital of Xiamen University, Xiamen, China; ^2^ The Department of Neuroscience, Institute of Neurosurgery, School of Medicine, Xiamen University, Xiamen, China; ^3^ The School of Clinical Medicine, Fujian Medical University, Fuzhou, China

**Keywords:** ferroptosis, autophagy, glioma, prognosis, risk model

## Abstract

**Background:** Ferroptosis plays an important role in glioma and significantly affects the prognosis, but the specific mechanism has not yet been elucidated. Recent studies suggest that autophagy regulates the process of ferroptosis. This study aimed to find potential autophagy-ferroptosis genes and explore the prognostic significance in glioma.

**Methods:** Ferroptosis and autophagy genes were obtained from two online databases (zhounan.org/ferrdb and autophagy.lu/). The RNAseq data and clinical information were obtained from the Chinese Glioma Genome Atlas (CGGA) database (http://www.cgga.org.cn/). Univariate, multivariate, lasso and Cox regression analysis screened out prognosis-related genes, and a risk model was constructed. Receiver operating characteristic (ROC) curve analysis evaluated the predictive efficiency of the model. Finally, a nomogram was constructed to more accurately predict the prognosis of glioma.

**Results:** We developed a Venn diagram showing 23 autophagy-ferroptosis genes. A total of 660 cases (including RNA sequences and complete clinical information) from two different cohorts (training group *n* = 413, verification group *n* = 247) of the CGGA database was acquired. Cohorts were screened to include five prognosis-related genes (*MTOR*, *BID*, *HSPA5*, *CDKN2A*, *GABARAPLA2*). Kaplan-Meier curves showed that the risk model was a good prognostic indicator (*p* < 0.001). ROC analysis showed good efficacy of the risk model. Multivariate Cox analysis also revealed that the risk model was suitable for clinical factors related to prognosis, including type of disease (primary, recurrence), grade (III-IV), age, temozolomide treatment, and 1p19q state. Using the five prognosis-related genes and the risk score, we constructed a nomogram assessed by C-index (0.7205) and a calibration plot that could more accurately predict glioma prognosis.

**Conclusion:** Using a current database of autophagy and ferroptosis genes, we confirmed the prognostic significance of autophagy-ferroptosis genes in glioma, and we constructed a prognostic model to help guide treatment for high grade glioma in the future.

## Introduction

Glioma is the most common primary malignant tumor in the brain. Current treatments for glioma include surgical resection, chemotherapy, radiotherapy, immunotherapy, and electric field therapy. Although many treatments exist, prognosis has not significantly improved (Duffau and Taillandier, 2015; Tan et al., 2020). Traditionally, gliomas have been divided into grades I-IV in pathological classification, of which grades I-II belong to low grades, and grades III-IV belong to high grades. According to the latest WHO classification in 2016, molecular pathology is now included in the classification of gliomas (Louis et al., 2016; White et al., 2020). This change shows the importance of molecular pathology for the diagnosis and treatment of glioma. Currently, molecular markers that affect prognosis have been identified for gliomas, but no exact and efficient target for clinical application yet exists.

Studies have found that ferroptosis plays an important role in nervous system tumors and notably affects the prognosis of gliomas (Buccarelli et al., 2018; Wan et al., 2021). Ferroptosis was initially described as a unique type of regulated cell death that is, observed in oncogenic *RAS*-mutated cancer cells and that is, distinct from apoptosis, necrosis, and autophagy at the morphological, biochemical, and genetic levels (Dixon et al., 2012; Xie et al., 2016). However, increasing evidence challenges these early observations and suggests that the autophagic machinery, at least under certain conditions, contributes to ferroptosis.

*BECN1* is a key regulator of autophagy. The *BECN1-SLC7A11* complex directly inhibits the activity of systemXc (-) and promotes ferroptosis (Kang et al., 2018). *Nrf2* is a key anti-ferroptosis transcription factor in liver cancer, and it can inhibit ferroptosis induced by sorafenib and erastin through the *p62-keap1-Nrf2* pathway (Sun et al., 2016). *RAB7A* can mediate the degradation of lipid droplets by the lipophagy pathway, increase the level of intracellular lipids, promote lipid peroxidation, and promote the ferroptosis of liver cancer cells induced by *RSL3*. Knockdown of the *RAB7A* gene inhibits lipophages and the degradation of lipid droplets, which can reverse *RSL3*-induced cell ferroptosis (Bai et al., 2019). *NCOA4* targets ferritin to the lysosome for autophagic degradation, increases unstable iron levels in cells, promotes reactive oxygen species production, and leads to cell ferroptosis. Knockout of the *NCOA4* gene inhibits ferritin autophagy, alleviates iron overload, and reverses erastin-induced cell ferroptosis (Mancias et al., 2014; Mancias et al., 2015).

However, the relationship between autophagy and ferroptosis in tumors is complex; Autophagy not only promotes but also inhibits ferroptosis. Adjusting autophagy activity to promote ferroptosis of tumor cells is of great significance to cancer treatment. Little research on the autophagy-ferroptosis connection in glioma has been conducted. In this study, we screened autophagy-ferroptosis genes using RNA sequences and clinical data in the Chinese Glioma Genome Atlas (CGGA) database. The purpose of this study was to investigate and verify the expression characteristics of autophagy-ferroptosis genes to predict the prognosis of glioma. This study also established a new predictive nomogram for related genes to more accurately assess the prognosis of glioma. These selected genes also provide a basis for subsequent research.

## Materials and Methods

### Patient Data

**FIGURE 1:**
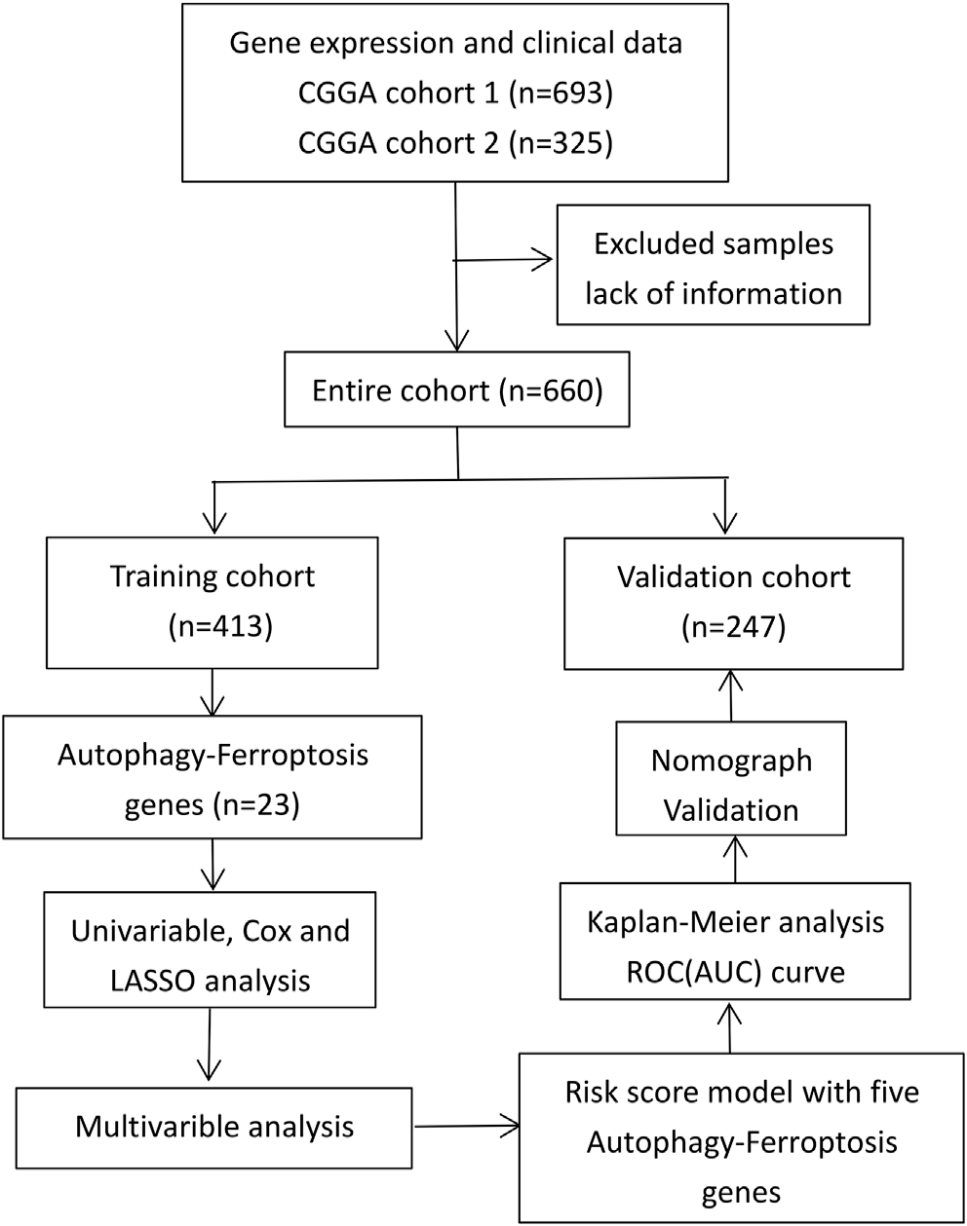
Study process.

The RNAseq data and clinical information of a training group and a verification group were obtained from the CGGA database (http://www.cgga.org.cn/); all data were complete and uniform (Zhao et al., 2017). We normalized gene expression by the RPKM (transcriptome reads per kilobase reads per million reads) method (Mortazavi et al., 2008). The study process is shown in Figure 1.

### Construction and Verification of Risk Models

Univariate and lasso regression analysis screened for the best prognosis-related genes with *p* < 0.01. A prognostic risk model for predicting overall survival (OS) was established. Patients with glioma were divided into high and low expression groups according to their median expression levels of prognosis-related genes. Kaplan-Meier survival analysis evaluated the relationship between the expression levels of prognostic-related genes and OS. Then, the correlations between the risk model and clinical characteristics were analyzed.

### Enrichment Analysis and Protein-Protein Interaction Network Construction

Gene Set Enrichment Analysis (GSEA) was used to enrich signal pathways between the low- and high-risk groups of patients with glioma (Subramanian et al., 2005). The cutoff criteria were a |normalized enrichment score (NES)| > 1.5 and a nominal *p* < 0.05. A protein-protein interaction (PPI) network of autophagy-ferroptosis genes was constructed to understand the relationship among genes.

### Construction and Verification of Nomogram

A nomogram was constructed according to the five prognosis-related genes to more accurately predict the prognosis for patients with glioma (Iasonos et al., 2008). This constructed allowed investigation of the 1-, 3-, 5-, 7-, and 9-years survival rates of patients with glioma. The concordance index (C-index) was calculated, and a calibration curve was plotted to assess the discrimination and accuracy of the nomogram.

### Statistical Analysis

All statistical analysis were performed with R (version 3.63, http://www.r-project.org/).

## Results

### Patient Database

Data from 660 patients with glioma were collected after missing values (RNAseq data and clinical characteristics) were excluded. Of this total, data from 413 patients were collected as a training group, and data from 247 patients were collected as a verification group. The clinical characteristics of the two cohorts are shown in Table 1.

**TABLE 1:** Clinical characteristics.

Clinical characteristics	Training group (*n* = 413)	Verification group (*n* = 247)	*P*
Age			
< 50	294	164	0.2
≥ 50	119	83	
Gender			
Female	182	98	0.27
Male	231	149	
Type			
Primary	248	194	< 0.05
Recurrence	165	53	
Grade			
II	47	81	< 0.05
III	87	64	
IV	279	102	
*IDH* mutation			
Yes	228	125	0.25
No	185	122	
1p19q codeletion			
Yes	87	50	0.8
No	326	197	
MGMT			
Yes	244	125	0.03
No	169	122	
Radiation therapy			
Yes	328	198	0.82
No	85	49	
TMZ therapy			
Yes	324	158	< 0.05
No	89	89	

### Construction and Verification of Risk Models

**FIGURE 2:**
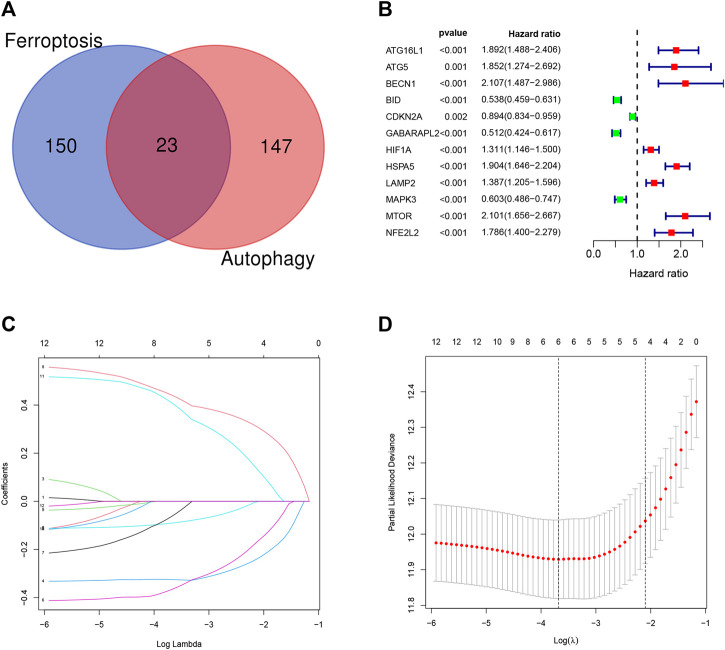
**(A)** Venn diagram showing that 23 genes have dual functions of autophagy and iron death. **(B–D)** Univariate and lasso regression screened the best prognosis-related genes.

**FIGURE 3:**
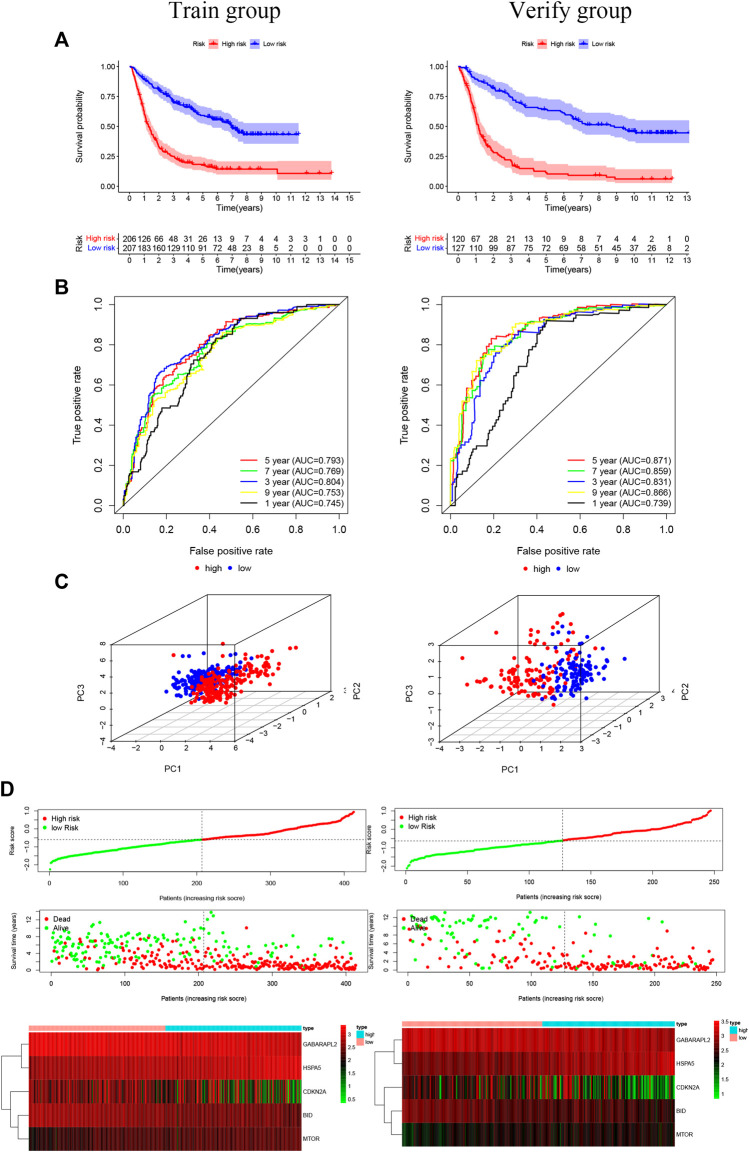
**(A)** The risk model of the experimental group and the validation group (*p* < 0.001). **(B)** The receiver operating characteristic curve suggests that the risk model has good short-term and long-term predictive values between the training and verification groups. **(C)** Component analysis suggests that the model can accurately distinguish high- and low-risk groups. **(D)** The risk curve and risk status show the survival status of the patient as the score increases. The risk heat map shows the expression of prognosis-related genes in the high- and low-risk groups.

**FIGURE 4:**
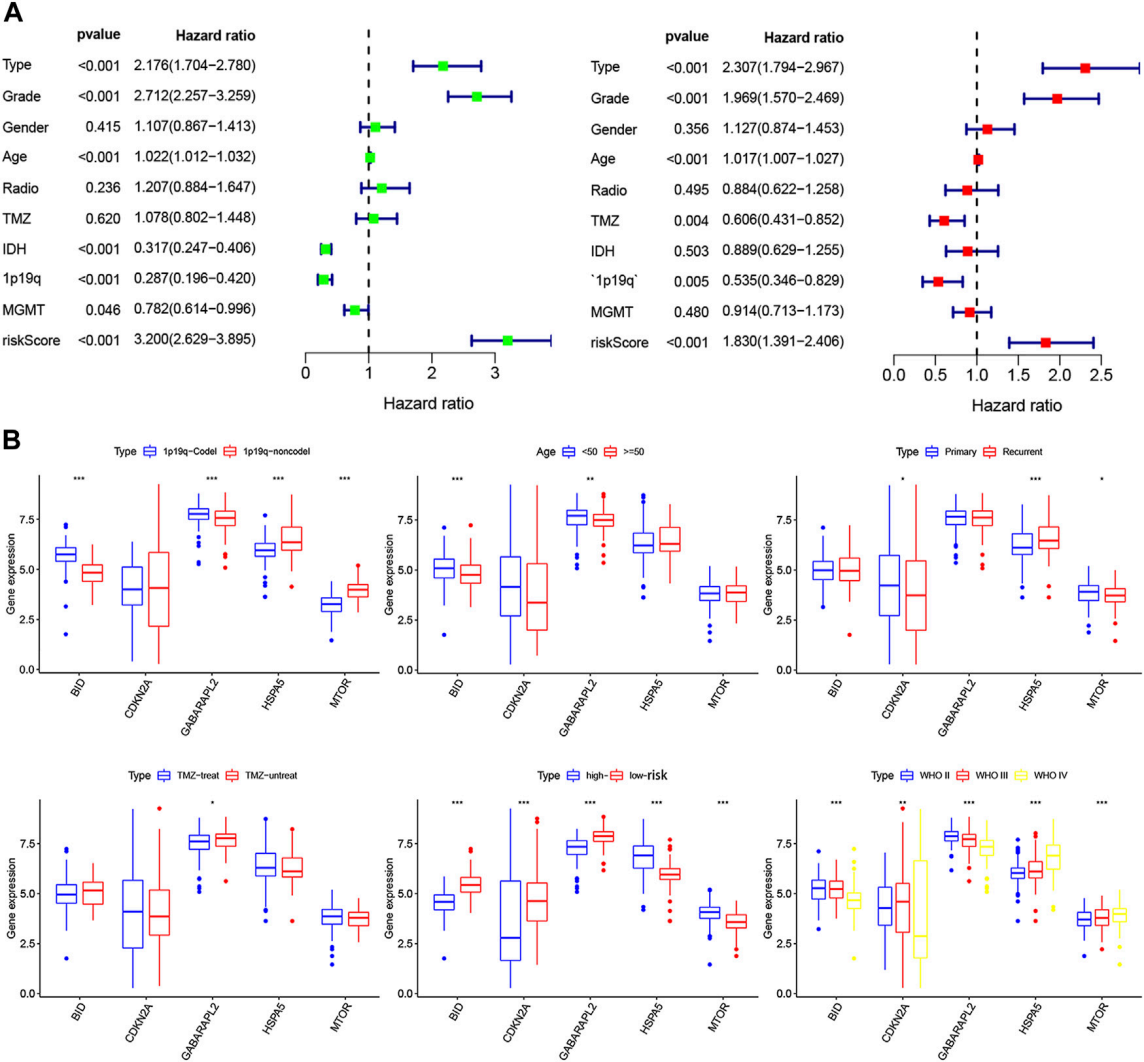
**(A)** Univariate and multivariate analyses screened clinical factors related to prognosis.**(B)** The relationship between the expression of five prognosis-related genes and clinical characteristics (*p < 0.05, **p < 0.01, ***p < 0.001).

**FIGURE 5:**
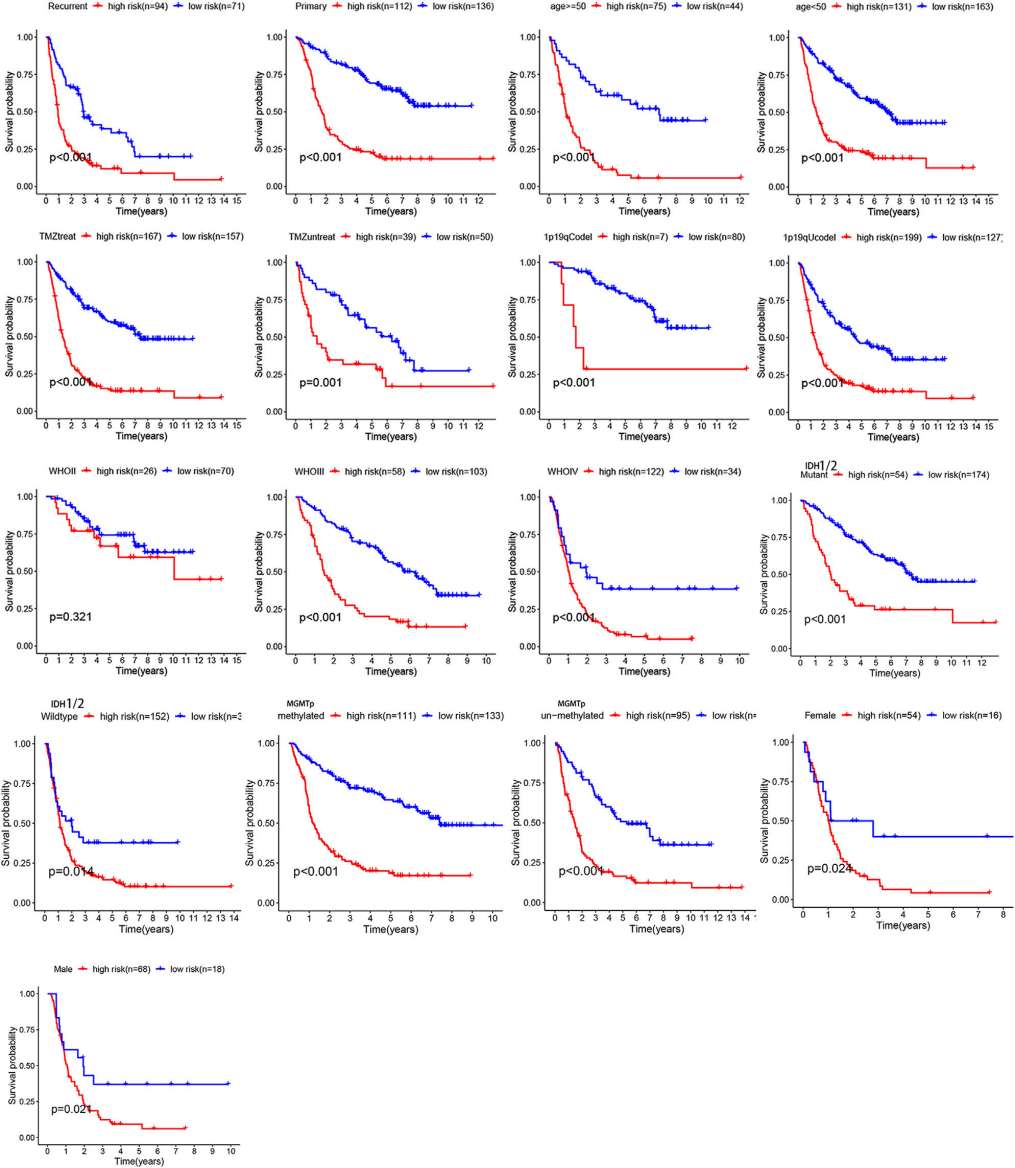
The relationship between risk model and clinical characters. The model is not applicable to WHO grade II glioma (*p* > 0.05).

Overall, 147 autophagy genes were obtained from an online database (http://www.autophagy.lu/) (Wang et al., 2019), and 150 ferroptosis genes were obtained from an online database (http://www.zhounan.org/) (Zheng et al., 2021). A constructed Venn diagram displayed 23 autophagy-ferroptosis genes (Figure 2A). Univariate and lasso regression analysis screened for five prognosis-related genes (*MTOR*, *BID*, *HSPA5*, *CDKN2A*, and *GABARAPLA2*) (Figures 2B–D). The risk score was defined as 0.40 × *HSPA5* + 0.34 × *MTOR* − 0.33 × *BID* − 0.08 × *CDKN2A* − 0.33 × *GABARAPLA2*. Kaplan-Meier survival analysis using the survival package estimated the associations between the expression levels of the prognosis-related genes and OS (Figure 3A). The prognostic performance was evaluated using time-dependent receiver operating characteristic (ROC) curve analysis within 1, 3, 5, 7, and 9 years to evaluate the predictive accuracy of the prognostic model (Figure 3B). The risk curve and risk status, risk heat maps, risk scatter plots, and PCA were drawn to evaluate the model’s ability to distinguish between high- and low-risk groups (Figures 3C,D). Then, the relationship between risk models and clinical characteristics was explored. Type, grade, age, temozolomide use, and 1p19q state were the risk factors associated with prognosis in glioma (Figure 4A). This risk model is applicable to these clinical risk factors except WHO grade II (Figure 5). The expression of the five prognosis-related genes was explored in relation to different clinical characteristics as well (Figure 4B).

### GSEA Enrichment Analysis and PPI Construction

**FIGURE 6:**
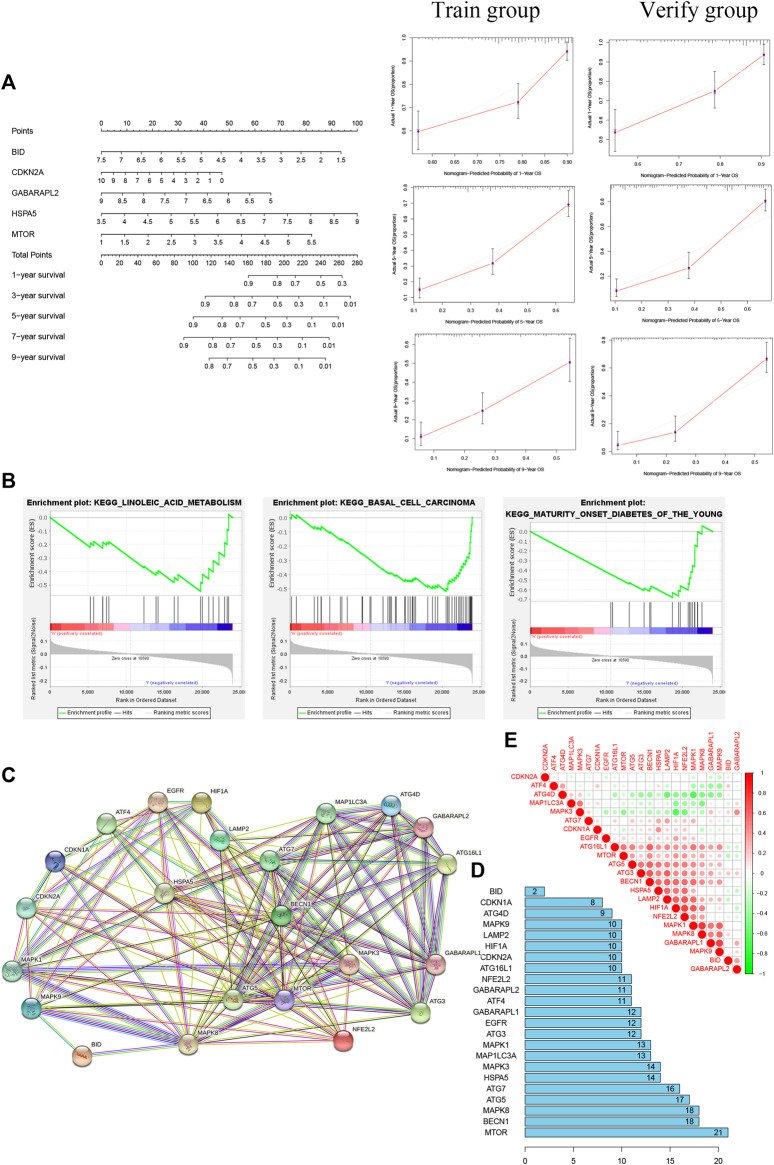
**(A)** Nomogram based on five prognosis-related genes (left) and calibration curves for 1, 5, and 9 years (right). **(B)** The main pathway enrichments were in basal cell carcinoma, linoleic acid metabolism, and mature-onset diabetes of the young. **(C, D)** Protein-protein interaction network suggesting that *MTOR* has the most extensive links. **(E)** Positive and negative correlations among 23 autophagy-ferroptosis genes.

Kyoto Encyclopedia of Genes and Genomes (KEGG) pathway analysis showed no significant enrichment in the high-risk group (*p* > 0.05); in the low-risk group, the main pathways of enrichment were in basal cell carcinoma, linoleic acid metabolism, and mature-onset diabetes of the young (Figure 6B). *MTOR* had the strongest correlation of 23 autophagy-ferroptosis genes in PPI (Figures 6C–E).

### Prognosis of Different Glioma Subgroups

**FIGURE 7:**
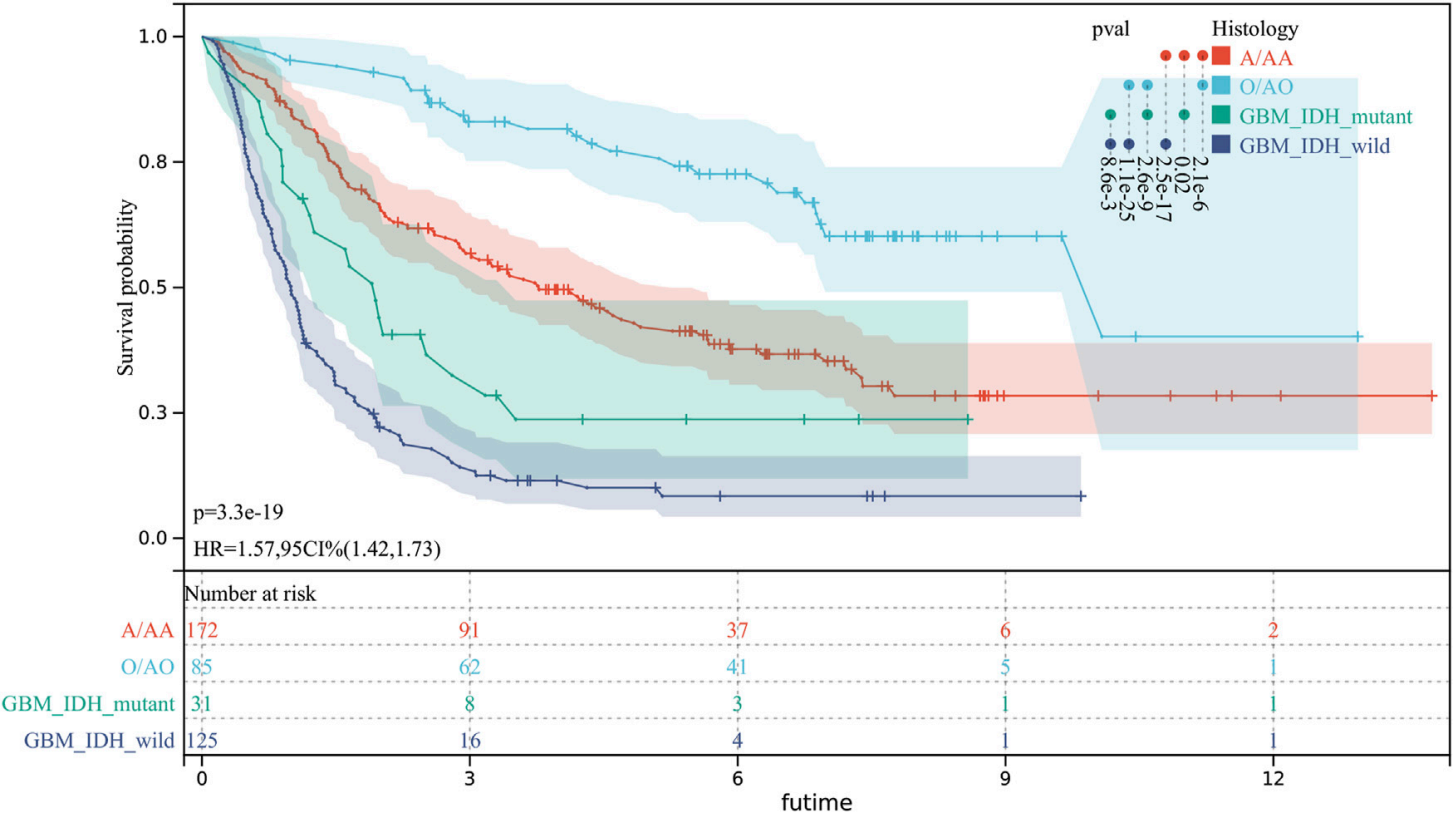
Prognostic differences in 4 glioma subgroups. GBM IDH wildtype; GBM IDH mutant; O/AO (Oligodendroglioma II/Anaplastic oligodendrocytoma III); A/AA (Astrocytoma II/Anaplastic astrocytoma III).

**FIGURE 8:**
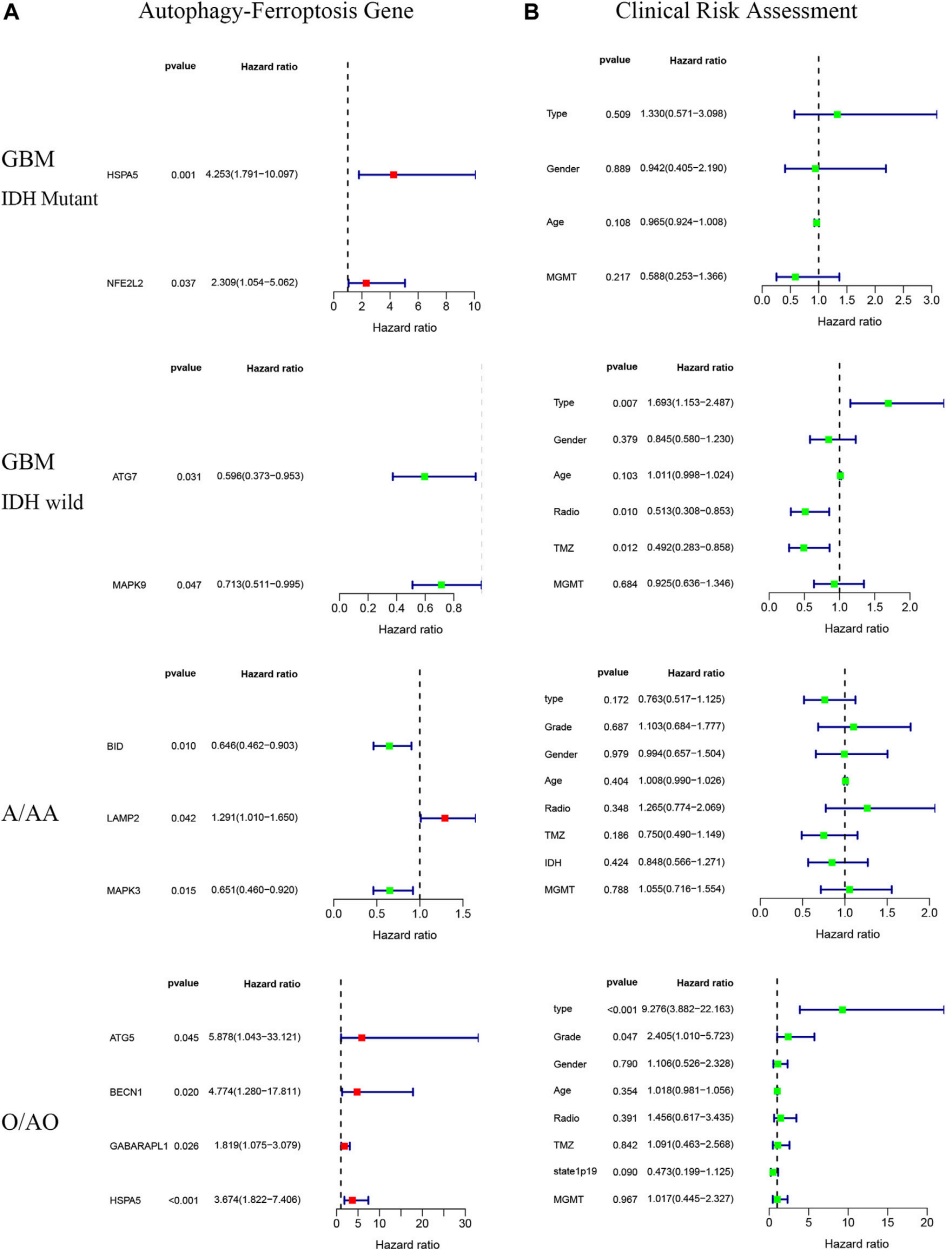
Autophagy-ferroptosis prognostic gene **(A)** and clinical risk assessment **(B)** in different subgroups of glioma.

There were significant prognostic differences in 4 glioma subgroups (GBM IDH wildtype, GBM IDH mutant, Oligodendroglioma II-III, Astrocytoma II-III) (Figure 7). 15 autophagy-ferroptosis genes were analyzed (Figure 8). The prognostic genes of GBM IDH mutant were HSPA5 and NFE2L2; GBM IDH wildtype were ATG7 and MAPK9; A/AA group includes BID, LAMP2 and MAPK3; ATG5, BECN1, GABARAPL1 and HSPA5 were prognostic genes in the O/AO subgroup; In the clinical risk assessment (Figure 8), Type (primary/recurrence), radiotherapy, and TMZ were prognostic factors for GBM IDH wildtype; Type (primary/recurrence) and Grade were prognostic factors for O/AO.

### Construction and Verification of Nomogram

A nomogram was built for accurately predicting 1-, 3-, 5-, 7-, and 9-years OS. The five prognosis-related genes were added to the prediction model (Figure 6A). The discrimination and accuracy of the nomogram were evaluated by C-index and calibration. The C-index was 0.72 in the training group and was 0.74 in verification group. Calibration curves of 1-, 5-, and 9-years survival rates were relatively close between prediction and observation.

## Discussion

Previous study explains the specific mechanism of ferroptosis in glioma. *ATF4* increases tumor angiogenesis and vascular structure shaping in an *xCT* activity-dependent manner, and downregulating *ATF4* expression can enhance the sensitivity of nerve tumor cells, which control the proliferation and vasculature of tumors, to ferroptosis (Chen et al., 2017). The overexpression of *Nrf2* and the knockout of *Keap1* can promote the proliferation and migration of tumor cells by upregulating the activity of *xCT*, thus changing the tumor microenvironment and inhibiting ferroptosis (Fan et al., 2017). The combined use of temozolomide and ferroptosis inducers can improve the therapeutic effect on glioma cells (Sehm et al., 2016). However, the mechanism of ferroptosis remains unclear. Although ferroptosis differs from other types of regulated cell death, studies have found that autophagy can regulate the process of ferroptosis. Few studies on autophagy-ferroptosis in gliomas have been conducted. In one study, amentoflavone treatment led to reduced cell viability and cell death by triggering ferroptosis in an autophagy-dependent manner in glioma (Chen et al., 2020). Additional study of autophagy-ferroptosis may provide new concepts to treat glioma in the future.

**FIGURE 9:**
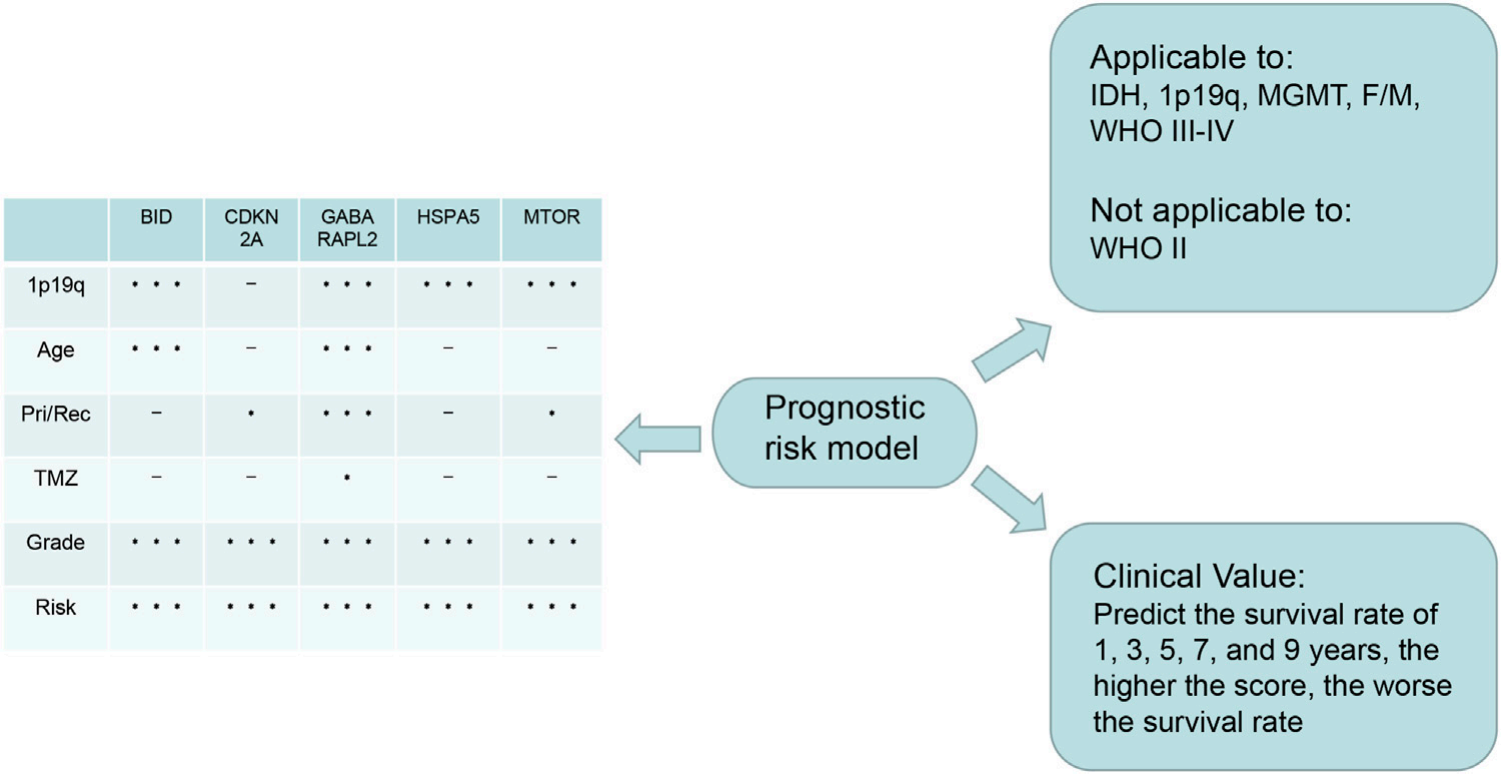
Summary: The lower right is the application value of the model, which can predict the 1, 3, 5, 7, and 9-years survival rate; The upper right is the scope of application of the model, which can be applied to IDH1 mutation/wild type, MGMT methylation/unmethylation, 1p19q deletion/no deletion, female/male, WHO III-IV glioma, except WHO II glioma; On the left is the expression of the five genes that constructing the model in different clinical characters. The express difference can provide a direction for future basic study.

In this study, we first identified 23 genes with dual functions of autophagy and ferroptosis. We designated these genes as autophagy-ferroptosis genes to distinguish them from the autophagy-dependent ferroptosis pathway, and because the relationship between the autophagy and ferroptosis genes has not been elucidated in previous studies, to our knowledge. We conducted various methods of statistical analysis, such as lasso regression, Cox regression, ROC curve analysis, and GSEA. We found that the risk model constructed by autophagy-ferroptosis genes are independently related to glioma prognosis. Summarized in Figure 9. Our model can improve the therapeutic effect of TMZ and the prognosis of high-grade gliomas, except for low-grade gliomas. Among the prognostic-related molecular subtypes included in the 2016 WHO guidelines, IDH wild type, MGMT unmethylated, and 1p19q no deletion showed poor prognosis. The model can also significantly improve the prognosis in these subtypes. This is a very promising result and provides a direction for future multi-target research. The disadvantage is that other molecular markers such as p53, TERT, EGFRv III, miR-181d etc can not be analyzed due to lack of relevant data in CGGA. Further exploring the expression differences of each gene, it is found that the expression differences of all genes are significantly related to the tumor grade, especially in grade IV gliomas, which further enhances the application value of the model in high-grade gliomas. In the high- and low-risk groups of the model, all genes also show expression differences, which implies that the selected genes are credible, because the expression differences gene are the basis for studying the pathogenic mechanism. Previous literature reported that gender differences in glioblastoma, estrogen and testosterone can affect the tumor microenvironment and thus change the prognosis, but our model has good predictive value in female and male without the discrepancy. The mechanism to overcome this gender difference is still need further research. With the continuous discovery of new molecular markers and the clinical application of new technologies such as immunotherapy and viral therapy, it is unclear whether the new theory will affect the predictive value of the model.

We identified five autophagy-ferroptosis genes related to prognosis: *MTOR*, *BID*, *HSPA5*, *CDKN2A*, and *GABARAPLA2*. In the previous literature, both the autophagy inducer rapamycin and the ferroptosis activator *RSL3* blocked *MTOR* activation and caused *GPX4* protein degradation in human pancreatic cancer cells, *GPX4* depletion enhances the anticancer activity of rapamycin and *RSL3 in vitro* or *in vivo*. In gestational diabetes, upregulated *SIRT3* enhanced autophagy activation by promoting the *AMPK-mTOR* pathway and decreasing *GPX4* levels to induce ferroptosis in trophoblastic cells (Han et al., 2020; Liu et al., 2021). Although studies involving *BID* have focused on autophagy and ferroptosis individually (Lamparska-Przybysz et al., 2006; Yang et al., 2013; Oppermann et al., 2014; Neitemeier et al., 2017), it remains unclear whether *BID* is involved in an autophagy-dependent ferroptosis pathway. *HSPA5* has inhibited autophagy and ferroptosis separately in previous studies (Cerezo and Rocchi, 2017; Zhu et al., 2017; Chen et al., 2019). Studies with *CDKN2A* and *GABARAPLA2* have not yet been reported. Basic biology requires experiments (*in vivo* or *in vitro*) and clinical studies to verify the functional characteristics of these genes.

## Conclusion

Using databases of autophagy and ferroptosis genes, we explored the prognostic significance of autophagy-ferroptosis genes in glioma and constructed a prognostic model to help improve care for patients with high grade glioma in the future.

## Data Availability

The original contributions presented in the study are included in the article/Supplementary Material, further inquiries can be directed to the corresponding author.
